# Reconstruction of a Risk Analysis Tool That Uses Nursing Data for Frailty Assessment Among Older Adults: Derivation and Internal Evaluation Study

**DOI:** 10.2196/67888

**Published:** 2025-12-02

**Authors:** Brian G Celso, Sarah E Ser, Urszula A Snigurska, Laurence M Solberg, Laurie J Duckworth, Elizabeth M Dunn, Mattia Prosperi, Robert J Lucero, Ragnhildur I Bjarnadottir

**Affiliations:** 1 Department of Surgery, UF Health Jacksonville, Jacksonville, FL, United States; 2 Department of Epidemiology, College of Public Health and Health Professions and College of Medicine, University of Florida, Gainesville, FL, United States; 3 College of Nursing, University of Florida, 1225 Center Drive, PO Box 100197, Gainesville, FL, 32610, United States, 1 3522736508, 1 3522736505; 4 Geriatrics Research, Education, and Clinical Center, North Florida/South Georgia Veterans Health System, Veterans Health Administration, Gainesville, FL, United States; 5 Clinical and Translational Science Institute, University of Florida, Gainesville, FL, United States; 6 Department of Epidemiology, College of Public Health and Health Professions, University of Florida, Gainesville, FL, United States; 7 School of Nursing, UCLA, Los Angeles, CA, United States

**Keywords:** frailty, risk assessment, nursing, clinical informatics, geriatrics

## Abstract

**Background:**

Frailty screening for older adults is of particular importance for those with declining health and social risk factors. However, many existing frailty tools do not offer automated appraisal in clinical settings due to challenges in data collection and the complexity of current approaches. Although routine frailty screening is inconsistently implemented, elements of frailty are captured in the electronic health record (EHR) from hospital admissions data. Thus, further adjustments and adaptations are required to correctly identify frailty.

**Objective:**

This study investigated existing frailty-related EHR data at two hospitals within a single health system to (1) identify key data elements and establish their availability in existing clinical workflows, and (2) use these data elements to reconstruct a validated and widely used frailty index.

**Methods:**

This developmental study included encounters of older adult patients (aged ≥65 years) admitted to medical-surgical units at 2 academic hospitals in North Florida between January 2012 and May 2021. EHR data were used to reconstruct a frailty index modeled after the Risk Analysis Index. Optimal cut points for frailty classification were determined through receiver operating characteristic analysis. Multiple logistic regression models were compared to evaluate predictive performance for hospital mortality, and component importance was assessed by sequentially removing each frailty parameter from the comprehensive model. Age-stratified analyses were performed to evaluate the robustness of classifications across age groups and race/ethnicity. All regression models were estimated to address rare outcome events.

**Results:**

A total of 10,863 hospital patients (45.5% male; mean age 75.4, SD 7.7 years) were included. Using optimal cut points, patients were classified as not frail (40.6%), prefrail (43.5%), or frail (14.9%), with corresponding mortality rates of 0.45%, 0.82%, and 3.24%. After adjustment for confounders, frail patients had significantly higher odds of mortality compared to not frail patients (odds ratio 7.14, 95% CI 4.30‐11.8; *P*<.001). Component importance analysis identified shortness of breath (area under the receiver operating characteristic curve reduction: 0.051) and functional status (area under the receiver operating characteristic curve reduction: 0.047) as the strongest predictors. Age-stratified analyses confirmed the classification system’s validity, with frail patients aged 65 to 75 years (mortality 3.97%) having higher mortality risk than not frail patients aged ≥86 years (mortality 0.48%).

**Conclusions:**

This study developed and validated a 3-category frailty classification system with strong predictive validity for hospital mortality among older adults. The generated frailty index leverages existing EHR data to capture frailty without increasing provider workload or interfering with workflow. Component importance analysis identified respiratory dysfunction and functional limitations as key predictors. This automated approach to frailty assessment could improve risk stratification, providing critical information to clinical teams with minimal burden and supporting clinical decision-making for hospitalized older adults. Future study of the overall reliability and validity of these derived frailty scores is warranted.

## Introduction

Frailty, a geriatric syndrome characterized by increased vulnerability to adverse health outcomes, poses a significant challenge in the current health care system [1]. Recent guidelines from both the American Academy of Family Physicians and the International Conference of Frailty and Sarcopenia Research emphasize the importance of widespread screening for adults aged ≥65 years, particularly those with specific health conditions or social risk factors [2]. Routine frailty screening has been used in some preoperative settings [3]; however, it has been sporadic and inconsistently implemented in most acute care settings. Therefore, there is a need to improve and systematize the process of identifying frailty in these clinical settings.

Numerous tools have been developed to assess frailty. Some, such as the frailty phenotype [4], focus on diagnosing frailty, whereas others, such as the Frailty Index [5] and the Risk Analysis Index (RAI) [6,7], aim to identify risk factors for frailty and assess the risk of adverse events such as mortality, disability, or hospital readmissions. However, agreement among different frailty tools is only fair to moderate [8]. Despite the availability of validated tools, their implementation within acute care settings remains limited. This is largely due to challenges related to data collection and the complexity of existing approaches. The key challenge involves the reliance of many existing frailty assessment tools on primary assessments that are time-consuming and may not be feasible in acute care settings, especially with critically ill patients [9]. These tools often require data beyond routine documentation, which can be burdensome for already overworked health care professionals. Any future frailty assessment tool must easily integrate with the routine clinical workflow to facilitate the uptake of the tool by health care professionals.

In the context of modern digitalized collection of health data, frailty assessment in acute care settings can be facilitated by capturing the data that are already routinely collected as part of administrative operations and clinical workflows [10]. For example, in the United States, frailty is captured as a diagnosis under the Centers for Medicare and Medicaid Services. Kim et al [11,12] showed that a Medicare claims-based frailty index predicted disability, mobility impairment, recurrent falls, and number of skilled nursing facility days. Despite support for the validity of a claims-based frailty index, the utility of claims-based tools is limited to retrospective analyses given the delay in data availability. Among older adults in particular, an accurate and reliable way to assess frailty is essential to meet the projected demographic shifts in both the number and proportion of world population that are caused by increased longevity [13] and for the US context, the transition of baby boomers to retirement age [14]. This limitation is particularly problematic in view of the increasing emphasis on precision or personalized medicine and the need for rapid clinical decision-making in real or near real time among hospitalized patients.

Owing to these modern information technology demands and demographic pressures, a novel, digital tool is needed to assess the risk of frailty in hospitalized older adults. Therefore, any future frailty assessment tool must leverage the readily available data from electronic health records (EHRs) that capture patient data for 84% of non–federal acute care hospitals in the United States [15]. EHRs include a wide array of data about a patient and may be sufficient sources of all information that is necessary for a *hands-off* frailty assessment.

This study addresses the limitations of existing frailty assessment tools in acute care by offering a data-driven approach to frailty risk assessment. The long-term goal is to develop a prognostic index for frailty that automates the process of frailty risk assessment based on EHR data collected through routine acute care. The proposed tool is entitled “The Automated Frailty Index Tool” (A-FIT). A-FIT leverages concepts from existing, validated frailty assessment tools and operationalizes these concepts based on expertise from clinical practice. The primary aim of this research was to acquire accuracy and clinical decision support comparable to that of existing frailty index tools while substantially reducing assessment and documentation burdens. The findings will inform the development of a robust and clinically actionable frailty assessment and decision support tool for hospitalized older adults, contributing to the advancement of precision medicine within acute care.

Developing and validating a prediction model for a robust risk assessment tool is a multistep process [16-18]. For an EHR data-driven tool, this may involve substantial workflow assessments and multiple iterations of operationalization and calibration of predictors. This study serves as a critical first step in the development process. The objectives of this study were to investigate existing frailty-related EHR data at 2 hospitals within a single health system to identify key data elements and establish their availability in existing clinical workflows and to use these data elements to reconstruct a validated and widely used frailty index.

## Methods

### Study Design

This was a development study that used secondary data from a large retrospective observational study of EHR data. The study included encounters of adult patients (aged ≥65 years) admitted to a medical/surgical unit at 1 of 2 academic hospitals in North Florida between January 2012 and May 2021. The larger study examined iatrogenic conditions among older adults in the acute care setting [19].

### Ethical Considerations

This study was conducted under a University of Florida IRB protocol (IRB201900208) designated as exempt under Category 4(iii) for secondary research using identifiable health information. Under this determination, informed consent was waived in accordance with 45 CFR 46.104(d)(4)(iii) and HIPAA provisions for research (45 CFR 164.501 and 164.512[b]). All data were analyzed within a secure, HIPAA-compliant environment, with no direct patient contact and no identifiable information disclosed. Procedures adhered to institutional and national guidelines and the principles of the Declaration of Helsinki. Standard technical and administrative safeguards were in place to protect patient privacy and ensure compliance with institutional and federal data security requirements.

### Conceptual Framework

The first step in the development process was informed by the Practice-Based Knowledge Discovery for Comparative Effectiveness Research framework proposed by Lucero and Bakken [20]. The framework, grounded in the Quality Health Outcomes Model [21] and the Knowledge Discovery in Databases [22] process, guides the extraction of actionable insights from electronic clinical data. This framework was chosen for its iterative approach to engaging practice-based experts in informatics research. A practice-based framework was crucial for developing a frailty index that not only demonstrated statistical and computational rigor but also possessed clear clinical utility. To this end, an interdisciplinary team of clinical experts was convened, including nurses, physicians, and physical therapists, as part of an iatrogenic conditions task force established to conduct the large retrospective observational study described earlier.

### Analytic Sample

In alignment with existing frailty screening recommendations, the analytic sample for this study included only hospital patients aged ≥65 years. The original dataset consisted of 60,475 inpatients. Patients with missing data for any of the 11 frailty parameters, including patients without any documented assessments of 1 of the 4 ADLs or those for whom living status (independent or dependent) could not be clearly established from the recorded residence (eg,“country clinic,” “unable to assess,” and “homeless”) were excluded. Furthermore, 1 patient was excluded because their documented age was outside the plausible range for age (ie, ≥116 years). This can occur during admissions to the emergency room, for example, in the case of major traumatic injury, where information could not be collected directly from a patient or their family member. The resulting analytical sample consisted of 10,863 patients with complete data for all frailty parameters. Among these patients, 113 (1.04%) experienced in-hospital mortality, which served as the primary outcome for model development and validation.

### Frailty Assessment Tool Construction

The second step in the development of the A-FIT involved a collaboration with the clinical expert task force to identify variables that were considered the most clinically meaningful representations of the RAI parameters and were expected to be widely and consistently documented within the medical/surgical setting. Through feedback from the team of clinical experts, important considerations were given to the stated goal that the tool enabled continuous assessment of frailty status across hospital admissions. To that end, 11 RAI frailty parameters were identified by the clinical task force.

For the next phase of development, the RAI was selected for comparison because the tool has an established validity across both surgical and nonsurgical settings. The RAI was developed and validated within the United States Department of Veterans Affairs (VA) health care system by adapting the Minimum Data Set Mortality Risk Index–Revised [23]. The RAI includes 11 assessment variables addressing various demographic, physiological, cognitive, and functional domains. As such, the RAI includes a broader range of factors beyond the 5 criteria of the frailty phenotype [4], but less than the Frailty Index [5], which requires consideration of at least 30 clinical variables. The RAI emphasizes effective distinction between frail and nonfrail patients in terms of increased risk of posttreatment complications and mortality. While the RAI was originally developed as a postoperative frailty assessment tool, it has been adapted and validated in nonoperative settings and across multiple hospitals.

To date, 3 versions of the index have been developed. The Clinical RAI (RAI-C) is a prospective screening tool in the form of a 14-item questionnaire addressing 11 frailty parameters [6]. The 11 frailty parameters are sex, age, cancer, renal failure, heart failure, cognitive decline, unintentional weight loss, poor appetite, shortness of breath at rest, activities of daily living (ADLs), and residence other than independent living. The Administrative RAI is a retrospective version that derives the same 11 frailty parameters from data available in surgical quality data sets [7]. The most recent version, called Risk Analysis Index-International Classification of Diseases (RAI-ICD), extends the application of RAI beyond the surgical population using the *International Classification of Diseases (ICD), Tenth Revision, Clinical Modification*, codes [24]. The RAI tools have numerous strengths, most notably their demonstrated validity and feasibility in assessing frailty in diverse and predominantly nonfrail populations. However, none of them fulfills the proposed criteria of automated assessment, thus requiring further adjustment and adaptation.

Our study extended the application of RAI by developing and validating empirically derived cut points for frailty risk categories. While previous applications of RAI primarily focused on binary classification (frail vs not frail), our approach sought to identify meaningful thresholds for 3 distinct risk categories (ie, not frail, prefrail, and frail) to enhance clinical utility and risk stratification.

### Data Operationalization

To operationalize the 11 RAI frailty parameters identified by the clinical task force, the fourth step in the A-FIT development encompassed categorizing the 11 parameters. The first category comprised parameters assessed on admission that are unlikely to change during the hospitalization. The RAI parameters in this category were age, sex, and residence other than independent living. The second category contained parameters assessed throughout hospitalization, for example, once a day or once a shift. These included shortness of breath and ADLs. Finally, there were parameters that are assessed on admission and may be updated as a patient’s condition changes but not with regular frequency. These included unintentional weight loss and poor appetite, which are part of required nursing admission documentation but may not be assessed further unless directly relevant to the principal diagnosis or care plan. Similarly, current active medical diagnoses are documented on admission and only updated at discharge unless additional unexpected clinical problems emerge.

Informed by this practice-based data operationalization, the final step was to reconstruct a frailty assessment based on RAI parameters. Key data sources are listed in Textbox 1. While this study used retrospective data, an emphasis was placed on the use of data points available to clinicians at the point of care. Of the 11 frailty parameters, 7 had such accessible data points. Owing to limitations of available secondary data, the need to use administrative codes (ICD-9 and ICD-10) to represent cancer, heart failure, renal failure, and cognitive decline was necessary during this stage of development. Finally, hospital mortality was determined based on a discharge disposition of ‘expired.’

Textbox 1. **Automated Frailty Index Tool data operationalization.**

Single time point

- Age on admission
- Sex
- Residence other than independent living (intake information)

Several time points

- Shortness of breath (at least one “dyspnea at rest” under Respiratory Pattern in Simple or Complex Assessment Flowsheet)
- Activities of daily living: eating, grooming, toileting, and walking (Activity Measure for Post-Acute Care assessment tool [25,26])

Continuous

- Unintentional weight loss
- Poor appetite (nutrition screening and admission documentation)
- Hospital problems or active diagnoses: cancer, heart failure, renal failure, cognitive decline (identified by *International Classification of Diseases, Ninth Revision*, or *International Classification of Diseases*, *Tenth Revision*, codes)

### Statistical Analysis

#### Descriptive statistics

Descriptive statistics for each parameter, including frequency distribution (count and percent), central tendency (mean and median), and variability (SD), were generated following construction of the frailty assessment parameters as displayed in Textbox 1. A frailty score for each patient was calculated based on the scoring criteria proposed by Hall et al [6]. Differences between frailty categories were assessed using Kruskal-Wallis tests for continuous variables (age and ADL scores) and χ^2^ tests for categorical variables. For significant Kruskal-Wallis results, post hoc pairwise comparisons were performed using Dunn test with Bonferroni correction. For the multicategory race/ethnicity variable, post hoc χ^2^ tests were conducted with Bonferroni adjustment to identify specific between-group differences. In addition to statistical significance testing, standardized mean differences (SMDs) were calculated to assess the magnitude of differences between frailty categories, with SMD values >0.1, >0.3, and >0.5 considered to represent small, moderate, and large differences, respectively.

#### Frailty Classification

To determine optimal cut points for frailty classification, a data-driven approach was used. First, a binary cutpoint was identified using the Youden index (maximizing sensitivity+specificity–1) to distinguish frail from nonfrail patients. Subsequently, established data-driven cut points were established using receiver operating characteristic curve analysis and the Youden index to maximize discrimination. This approach yielded 3 clinically meaningful risk categories: not frail, prefrail, and frail. The robustness of these classifications was evaluated across age groups to ensure the index captured vulnerability beyond chronological age alone.

#### Model Development and Validation

To validate our frailty index and determine its incremental value beyond basic patient characteristics, several comparative models were constructed. The first model included only potential confounding factors (eg, hospital location, race/ethnicity, and smoking status). The second model added key clinical predictors (eg, age, sex, and functional status) to assess whether our composite frailty index provided additional predictive power beyond these established risk factors. The third and fourth models evaluated our frailty index in both continuous and categorical forms. Finally, a comprehensive model, including all individual frailty components, was constructed to determine whether our composite index adequately captured the predictive information contained in its constituent parts.

Multiple modeling approaches were compared to evaluate the predictive performance of the frailty index. Four models were constructed: (1) a confounders-only model, including hospital location, race/ethnicity, and smoking status; (2) a key components model adding age, sex, and functional status (ADL) to the confounders; (3) a continuous frailty index model adding the index score to the confounders; and (4) a categorical frailty index model adding the 3-category frailty classification to the confounders. Additionally, these models were compared to a comprehensive model, including all individual frailty components.

Model performance was evaluated using discrimination (AUC) and calibration (Akaike Information Criterion [AIC]). Likelihood ratio tests were used to compare nested models. To assess the contribution of individual components to the frailty index’s predictive ability, component importance analysis was conducted by sequentially removing each component and measuring the resulting change in AUC.

Additionally, age-stratified analyses were performed to evaluate the performance of the frailty classification system across different age groups (ie, 65‐75, 76‐85, and ≥86 years). For each age stratum, separate logistic regression models were fitted to estimate the odds of mortality associated with frailty categories, adjusting for potential confounders, including hospital location, race/ethnicity, and smoking status. All regression models were estimated using Firth’s penalized likelihood approach to address potential issues related to rare events, as in-hospital mortality was an uncommon outcome in our dataset. Analyses were completed using R (version 4.3.1 [25]) with packages, including logistf, pROC, dplyr, tidyr, cutpointr, and dunn.test.

## Results

### Sample Characteristics

A total of 10,863 hospital patients aged ≥65 years were included. The average age was 75.4 (SD 7.7) years. Of the included participants, 4938 (45.5%) were male. With regard to race/ethnicity, 7724 participants (71.1%) were non-Hispanic White, 2406 participants (22.1%) were non-Hispanic Black, 314 participants (2.9%) were Hispanic, and the remaining 419 participants (3.9%) were other or unknown race/ethnicity. Hospital mortality occurred for 113 patients in the sample (1.04%). The frailty index scores ranged from 24 to 84. The skewness statistic was 0.39 (SE 0.02), indicating mild positive skew. The overall mean frailty score for the sample was 45.3 (SD 8.7).

### Development of Frailty Categories

Using a data-driven approach, optimal cut points were identified to classify patients into 3 distinct risk categories. The first cutpoint at a frailty score of 54 was identified using the Youden index to maximize the sum of sensitivity (53.1%) and specificity (82.5%) for mortality prediction. A second cut point at a frailty score of 42 was established to distinguish between low-risk and intermediate-risk patients. On the basis of these thresholds, patients were classified as not frail (RAI <42), prefrail (RAI 42‐53), and frail (RAI ≥54). The distribution of patients across these categories was as follows: not frail 40.8% (n=4434), prefrail 43.8% (n=4763), and frail 15.3% (n=1666). Hospital mortality rates showed a clear gradient across categories: 0.45% (95% CI 0.28%–0.70%) in not frail, 0.82% (95% CI 0.59%–1.12%) in prefrail, and 3.24% (95% CI 2.49%–4.23%) in frail patients.

### Differences Between Frailty Categories

Significant differences were observed across frailty categories for both demographic and clinical characteristics and presented in Table 1. Post hoc pairwise comparisons using Dunn test with Bonferroni correction revealed significant age differences across all 3 frailty categories (all *P*<.001), with progressively higher median ages from not frail to prefrail to frail groups. For ADL scores, frail patients had significantly higher scores (indicating worse function) than both prefrail (*P*<.001) and not frail groups (*P*=.046), but no significant difference was found between not frail and prefrail groups (*P*=.12).

**Table 1.** Demographics based on frailty status.

	Overall	Not frail	Prefrail	Frail	SMD^a^	*P* value
n	10,863	4434	4763	1666
Age, mean (SD), years	75.4 (7.7)	72.0 (68.0, 77.0)	76.0 (70.0, 83.0)	77.0 (71.0, 84.0)	0.454	<.001
ADL^b^ score, median (interquartile range)	13.0 (12.0, 14.0)	13.0 (12.0, 14.0)	13.0 (11.0, 15.0)	13.0 (11.0, 14.0)	0.068	.002
Sex, n (%)					0.549	<.001
Female	5925 (54.5)	3084 (69.6)	2328 (48.9)	513 (30.8)		
Male	4938 (45.5)	1350 (30.4)	2435 (51.1)	1153 (69.2)		
Race/ethnicity, n (%)					0.065	.01
Hispanic	314 (2.9)	136 (3.1)	140 (2.9)	38 (2.3)		
Non-Hispanic Black	2406 (22.1)	917 (20.7)	1123 (23.6)	366 (22.0)		
Non-Hispanic White	7724 (71.1)	3193 (72.0)	3331 (69.9)	1200 (72.0)		
Other	419 (3.9)	188 (4.2)	169 (3.5)	62 (3.7)		
Hospital location, n (%)					0.08	<.001
Gainesville	8525 (78.5)	3497 (78.9)	3665 (76.9)	1363 (81.8)		
Jacksonville	2338 (21.5)	937 (21.1)	1098 (23.1)	303 (18.2)		
Dependent living, n (%)					0.427	<.001
No	10,101 (93.0)	4376 (98.7)	4383 (92.0)	1342 (80.6)		
Yes	762 (7.0)	58 (1.3)	380 (8.0)	324 (19.4)		
Shortness of breath, n (%)					0.505	<.001
No	10,184 (93.7)	4405 (99.3)	4488 (94.2)	1291 (77.5)		
Yes	679 (6.3)	29 (0.7)	275 (5.8)	375 (22.5)		
Weight loss, n (%)					0.523	<.001
No	9847 (90.6)	4328 (97.6)	4324 (90.8)	1195 (71.7)		
Yes	1016 (9.4)	106 (2.4)	439 (9.2)	471 (28.3)		
Poor appetite, n (%)					0.656	<.001
No	8694 (80.0)	4090 (92.2)	3733 (78.4)	871 (52.3)		
Yes	2169 (20.0)	344 (7.8)	1030 (21.6)	795 (47.7)		
Cancer diagnosis, n (%)					1.368	<.001
No	7849 (72.3)	4404 (99.3)	2998 (62.9)	447 (26.8)		
Yes	3014 (27.7)	30 (0.7)	1765 (37.1)	1219 (73.2)		
Heart failure, n (%)					0.49	<.001
No	8093 (74.5)	3855 (86.9)	3315 (69.6)	923 (55.4)		
Yes	2770 (25.5)	579 (13.1)	1448 (30.4)	743 (44.6)		
Renal failure, n (%)					0.788	<.001
No	7935 (73.0)	4035 (91.0)	3205 (67.3)	695 (41.7)		
Yes	2928 (27.0)	399 (9.0)	1558 (32.7)	971 (58.3)		
Cognitive decline, n (%)					0.272	<.001
No	9406 (86.6)	4139 (93.3)	3939 (82.7)	1328 (79.7)		
Yes	1457 (13.4)	295 (6.7)	824 (17.3)	338 (20.3)		
Mortality, n (%)					0.142	<.001
No	10,750 (99.0)	4414 (99.5)	4724 (99.2)	1612 (96.8)		
Yes	113 (1.04)	20 (0.5)	39 (0.8)	54 (3.2)		

a SMD: standardized mean difference.

b ADL: activities of daily living.

Analyses of race/ethnicity distribution using χ^2^ tests with Bonferroni correction identified a significant difference between non-Hispanic Black and non-Hispanic White patients (*P*=.039). Non-Hispanic Black patients were less likely to be classified as not frail (38.1% vs 41.3%) and more likely to be classified as prefrail (46.7% vs 43.1%) compared to non-Hispanic White patients. The proportion classified as frail was similar across all racial/ethnic groups, ranging from 12.1% among Hispanic patients to 15.5% among non-Hispanic White patients.

### Model Performance Comparison

Multiple models were compared to evaluate the predictive performance of our frailty index. A baseline model, including only potential confounders (eg, hospital location, race/ethnicity, and smoking status), showed poor discrimination for hospital mortality (AUC 0.592). A model incorporating key clinical predictors (eg, age, sex, and functional status) performed better (AUC 0.760). The continuous frailty index model demonstrated good discrimination (AUC 0.738), whereas the categorical frailty classification model achieved similar performance (AUC 0.717). A comprehensive model using all individual frailty components demonstrated the highest discrimination (AUC 0.887).

Likelihood ratio tests confirmed that both the continuous frailty index (χ²_1_=100.7; *P*<.001) and the 3-category classification (χ²_2_=75.4; *P*<.001) significantly improved prediction compared to the confounders-only model (Table 2). The AIC values showed a similar pattern, with the all-components model having the lowest AIC (935.47), followed by the continuous frailty index model (1142.02), the categorical model (1169.38), and the confounder-only model (1240.73).

**Table 2.** Frailty index model comparisons.

Model	AUC^a^	AIC^b^	AUC_CI	χ²	*P* value
Model A (confounders only)^c^	0.592	1240.7	0.542‐0.642	—^d^	—
Model B (key components)^e^	0.760	1252.3	0.713‐0.807	—	—
Model C (continuous frailty index plus confounders)	0.738	1127.8	0.689‐0.788	—	—
Model D^f^ (categorical frailty index plus confounders)	0.717	1239.5	0.665‐0.768	—	—
Model E (all components)	0.887	1142	0.853‐0.921	—	—
Confounders only	0.592	1245.2	0.542‐0.642	—	—
Key components	0.760	1169.4	0.713‐0.807	—	—
Continuous frailty index	0.738	1250.4	0.689‐0.788	100.7	<.001
Categorical frailty index	0.717	935.5	0.665‐0.768	75.4	<.001
All components	0.887	1234.8	0.853‐0.921	—	—

a AUC: area under receiver operating characteristic curve.

b AIC: calibration.

c Confounders (hospital location, race/ethnicity, and smoking status).

d Not applicable.

e Key components (age, sex, and activities of daily living).

f Categorical age groups (ie, 65-75, 76-85, and ≥86 years).

### Component Importance Analysis

**Figure 1.**
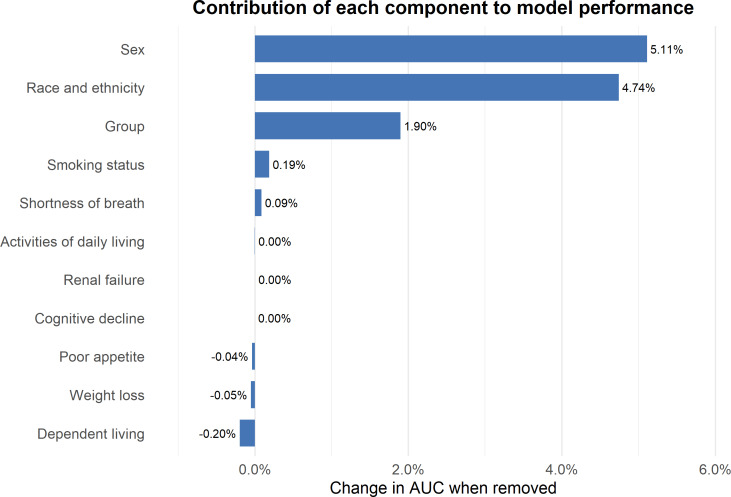
Contribution of each component to model performance. ADL: activities of daily living; AUC: area under receiver operating characteristic curve.

To identify the key drivers of predictive performance, a component importance analysis was conducted by sequentially removing each frailty parameter from the comprehensive model shown in Figure 1. This analysis revealed that shortness of breath was the strongest predictor (AUC reduction: 0.051 when removed), followed by ADL limitations (AUC reduction: 0.047) and renal failure (AUC reduction: 0.019). Interestingly, age showed minimal independent contribution within this 65+ population (AUC reduction: –0.0005), suggesting that our frailty index captures vulnerability beyond chronological age.

### Age-Stratified Analysis

As presented in Table 3, the proportion of patients classified as frail increased with age: 12% in the 65 to 75 years age group, 18% in the 76 to 85 years age group, and 26% in the ≥86 age group. In all age groups, the frail category identified patients at substantially higher risk, with mortality rates 6 to 8 times higher than patients classified as not frail. Notably, a frail patient in the 65 to 75 years age group (mortality 3.97%) had higher mortality risk than a not frail patient in the ≥86 years age group (mortality 0.48%).

**Table 3.** Age-stratified odds of mortality controlling for hospital location, race/ethnicity, and smoking status.

	Total (N)	Mortality, n (%)	Adjusted odds ratio	95% CI	*P* value
Age group (years)					
65‐75					
Not frail	3066	15 (0.49)	*Reference*	—^a^	—
Prefrail	2287	17 (0.74)	1.56	0.78‐3.13	.208
Frail	706	28 (3.97)	8.50	4.59‐16.32	<.001
76‐85					
Not frail	1159	4 (0.35)	*Reference*	—	—
Prefrail	1714	16 (0.93)	2.47	0.94‐8.01	.069
Frail	611	16 (2.62)	6.42	2.41‐21.02	<.001
≥86					
Not frail	209	1 (0.48)	*Reference*	—	—
Prefrail	762	6 (0.79)	1.15	0.23‐11.12	.88
Frail	349	10 (2.87)	3.99	0.91‐37.48	.07

a Not applicable.

After adjusting for hospital location, race/ethnicity, and smoking status, age-stratified logistic regression models showed varying strengths of association between frailty categories and mortality. In the 65 to 75 years age group, frail patients had significantly higher odds of mortality compared to not frail patients (odds ratio [OR] 8.50, 95% CI 4.59‐16.32; *P*<.001), whereas prefrail status showed a positive but nonsignificant association (OR 1.56, 95% CI 0.78‐3.13; *P*=.208).

In the 76 to 85 years age group, the association remained strong for frail patients (OR 6.42, 95% CI 2.41‐21.02; *P*<.001), but prefrail status did not reach statistical significance (OR 2.47, 95% CI 0.94‐8.01; *P*=.069). In the oldest age group (≥86 years), the point estimate for frail status remained elevated (OR 3.99, 95% CI 0.91‐37.48) but did not reach statistical significance (*P*=.07).

## Discussion

### Principal Findings

The purpose of this research was to automate risk assessment and early frailty detection by using readily available EHR data, with the long-term goal of improving frailty detection while minimizing the documentation burden on health care providers. Our study not only developed a viable frailty index but also established empirically derived cut points for a 3-category frailty classification system with strong predictive validity for hospital mortality. This work demonstrates that routinely collected EHR data can be leveraged to create a clinically meaningful risk stratification tool for older adult medical/surgical patients.

### Frailty Index Performance and Comparison With Previous Work

In this study, the average frailty index score was 45.3, considerably higher than the mean frailty scores reported in the development and validation of the original RAI instruments (11.8 for clinical RAI and 9.6 for RAI-administrative). This difference is largely explained by our focus on adults aged ≥65 years, whereas Hall et al [6] included all adults aged ≥18 years. Our 3-category classification system identified 14.9% of patients as frail (RAI ≥54), 43.5% as prefrail (RAI 42‐53), and 40.6% as not frail (RAI <42). This distribution aligns with previous literature, suggesting that approximately 10% to 15% of community-dwelling older adults are frail, with a larger proportion in the prefrail category [27,28].

Our model comparison revealed that while individual frailty components provide optimal predictive power (AUC=0.887), the composite frailty index (AUC=0.738) and 3-category classification (AUC=0.717) offer substantial improvement over basic demographic factors alone (AUC=0.592). Interestingly, a simpler model using only age, sex, and functional status achieved slightly better discrimination (AUC=0.760) than our composite index, echoing previous findings about the powerful predictive value of functional status in geriatric populations [29-31]. However, our 3-category frailty classification offers conceptual advantages, clinical interpretability, and standardization that may outweigh the modest reduction in statistical performance.

### Component Importance and Novel Insights

Our component importance analysis yielded several notable findings. First, respiratory dysfunction (shortness of breath) emerged as the strongest mortality predictor, with an AUC reduction of 0.051 when removed from the model. This aligns with growing recognition of respiratory parameters as important indicators of acute deterioration in hospitalized patients [32,33]. That shortness of breath was the most significant factor for the development of frailty, which is consistent with other research that revealed among older persons, respiratory impairment and frailty are strongly associated with one another [29,34]. Additionally, there is a substantial increase in vulnerability to adverse health outcomes, such as heart failure or chronic obstructive pulmonary disease, and risk of death when both are present. These conditions contribute to reduced physical activity, muscle weakness, and overall functional decline, thereby accelerating the progression toward frailty. Unfortunately, frailty is often underrepresented in respiratory management guidelines, which may contribute to limited recognition of its comorbidity in respiratory care [35].

Functional status (ADL) was the second most important component (AUC reduction: 0.047), consistent with extensive literature establishing functional measures as robust predictors of outcomes in older adults [36]. Notably, age contributed minimally to prediction beyond these clinical factors, confirming that our index captures frailty as a distinct clinical entity rather than merely reflecting chronological aging. This finding is particularly significant, given that our study focused exclusively on adults aged ≥65 years, suggesting that frailty assessment provides valuable risk stratification even within the older adult population.

### Age-Stratified Analysis and Demographic Patterns

Our age-stratified analysis revealed that while the prevalence of frailty increases with age (12% in ages 65‐75 years, 18% in ages 76‐85 years, and 26% in ages ≥86 years), the predictive value of frailty categories remains robust across all age strata. The striking finding that frail patients aged 65 to 75 years had higher mortality risk than nonfrail patients aged ≥86 years underscores the clinical importance of frailty assessment beyond simple age-based approaches to risk stratification.

The gradient in ORs across age groups (OR decreasing from 8.50 to 6.42 to 3.99) suggests that while frailty remains an important predictor of mortality across all age groups, its relative impact may diminish somewhat in the oldest old. This pattern aligns with the concept of survival bias, where those who reach advanced age despite being classified as frail may represent a resilient subpopulation [37]. Nevertheless, the consistency of the association pattern across age strata supports the clinical utility of the frailty classification system throughout the geriatric age spectrum.

The declining statistical significance observed in the oldest age group likely reflects limited statistical power rather than absence of effect, as evidenced by the wide CIs. This highlights a common challenge in geriatric research where sample sizes often decrease with advancing age categories.

Our analysis of race/ethnicity distribution revealed significant differences between non-Hispanic Black and non-Hispanic White patients, with Black patients less likely to be classified as not frail (38.1% vs 41.3%) and more likely to be classified as prefrail (46.7% vs 43.1%). This finding adds to the growing body of evidence on health disparities in frailty prevalence [38,39] and highlights the importance of considering social determinants of health in frailty assessment and intervention planning.

The ORs for mortality demonstrate the strong discriminative ability of our classification system; after adjusting for confounders, prefrail patients had 1.8 times higher odds of mortality than not frail patients, whereas frail patients had 7.1 times higher odds. These magnitudes of association are clinically meaningful and comparable to established frailty measures in the literature [4-7].

### Technical Implications

This study went beyond validation of an existing tool. Rather than using identical predictors and operational definitions from the original RAI development, the framework was adapted to work with routinely available EHR data, enhancing translatability across sites and settings. Our methodological approach to handling the rare outcome (hospital death) was rigorous, using Firth’s penalized likelihood method to address potential bias in logistic regression with rare events.

Our 3-category classification system represents an important methodological advancement over binary approaches. By identifying a prefrail intermediate category, our system provides greater nuance for risk stratification and potentially enables earlier intervention before patients progress to more severe frailty. The use of SMDs in addition to statistical testing helped identify clinically meaningful differences between frailty categories, particularly for functional and respiratory parameters.

### Clinical Implications

Frailty is recognized as an important predisposing factor for and predictor of adverse outcomes. Therefore, there is a necessity for frailty screening to identify potentially modifiable vulnerabilities among frail older adults. A practical application includes such procedures as the utilization of prehabilitation and optimized nutrition that have demonstrated beneficial effects on functional outcomes postoperatively [40-45]. This research extends these efforts beyond the surgical arena to also optimize hospitalized medical patients’ condition and reduce the incidences of many possible complications that increase morbidity, prolong lengths of stay, and may lead to mortality.

The clinical implications of our findings are substantial. First, our 3-category frailty classification system provides clinicians with a practical framework for risk stratification that can be automatically generated from EHR data without additional documentation burden. Second, the identification of shortness of breath and functional status as the strongest predictors suggests potential targets for intervention and monitoring. Third, our finding that frailty predicts mortality independent of age challenges age-based stereotypes and encourages individualized risk assessment.

Our tool extends frailty assessment beyond the surgical arena to all hospitalized medical/surgical patients, addressing a significant gap in current practice. While much previous research on functional decline due to frailty has focused on surgical outcomes [46-48], our work demonstrates the relevance of frailty assessment across the broader inpatient population. Furthermore, addressing shortness of breath through appropriate medical intervention and pulmonary rehabilitation can potentially mitigate the risk of frailty development in older adults for improved clinical outcomes [49,50].

Frailty is also appreciated as a dynamic condition for older adults, who may experience changes in their frailty status over time. This may occur as either a gradual decline or sudden change in an acute care setting or possibly reversible with rehabilitation. Previous research has shown an association between older age and faster progression of frailty [51]. Additionally, patients who were postsurgery, especially emergency general surgeries, can induce or worsen frailty. Ng et al [52] showed that 33.3% of older patients undergoing emergency surgery experienced an increase in their frailty status 90 days postoperatively. Furthermore, 15.9% of patients were considered with a new frail condition impacting their discharge destination. Our automated approach creates opportunities for continuous monitoring of frailty status across hospital admissions, potentially allowing clinical teams to detect and respond to changes in patient vulnerability during hospitalization.

### Strengths and Limitations

This study has several strengths, including the large sample size, rigorous methodological approach, and comprehensive validation of the frailty classification system. The use of data-derived cut points for our 3-category system provides a data-driven foundation for clinical implementation. Our component importance analysis identified key drivers of mortality prediction, offering valuable insights for focused assessment and intervention.

However, limitations must be acknowledged. First, our reliance on secondary data meant that some frailty parameters were derived from administrative codes rather than direct clinical assessment. Second, hospital mortality was a rare event (1.04%), necessitating specialized statistical approaches and potentially limiting statistical power for some analyses. Our use of Firth’s penalized likelihood method helped address issues related to rare events, but validation in cohorts with more outcome events would strengthen confidence in our findings.

Additionally, our analytical sample excluded patients whose residential status could not be clearly established, including homeless individuals, potentially introducing selection bias. Future studies should investigate the inclusion of homelessness as an independent risk factor to improve generalizability. The observed differences in frailty distribution between racial/ethnic groups also warrant further investigation into potential disparities in frailty assessment and care.

While our study demonstrated the predictive validity of our frailty classification for in-hospital mortality, longer-term outcomes or other clinically relevant end points, such as functional decline, discharge disposition, or readmission, were not assessed. Future research should explore these additional outcomes to further validate the clinical utility of our approach and include sequential measures to investigate how frailty changes during acute care so that clinicians can monitor and respond to changes in patient vulnerability during hospitalization.

### Conclusions

This paper describes the development and validation of a 3-category frailty classification system based on readily available EHR data that demonstrate strong predictive validity for hospital mortality among older adults. Our approach provides meaningful risk stratification with mortality rates of 0.45%, 0.82%, and 3.24% across not frail, prefrail, and frail categories, respectively. Component importance analysis identified respiratory dysfunction and functional limitations as the strongest predictors, offering potential targets for monitoring and intervention.

This automated approach to frailty assessment could provide critical information to clinical teams with minimal additional documentation burden, potentially improving risk stratification and clinical decision-making for hospitalized older adults. Further research should explore how this frailty classification system performs for additional outcomes and whether implementation in clinical workflows improves patient care and outcomes.

## Abbreviations

A-FIT: Automated Frailty Index Tool
ADL: activities of daily living
AUC: area under the receiver operating characteristic curve
EHR: electronic health record
ICD: International Classification of Diseases
OR: odds ratio
RAI: Risk Analysis Index
RAI-C: Clinical Risk Analysis Index
SMD: standardized mean difference
